# Mixed asexual and sexual reproduction in the Indo-Pacific reef coral *Pocillopora damicornis*

**DOI:** 10.1002/ece3.721

**Published:** 2013-08-22

**Authors:** David J Combosch, Steven V Vollmer

**Affiliations:** Marine and Environmental Sciences, Northeastern UniversityNahant, Massachusetts, 01908

**Keywords:** Evolutionary significance, minimal cryptic sex, mixed reproduction, parthenogenesis, pseudogamy

## Abstract

*Pocillopora damicornis* is one of the best studied reef-building corals, yet it's somewhat unique reproductive strategy remains poorly understood. Genetic studies indicate that *P. damicornis* larvae are produced almost exclusively parthenogenetically, and yet population genetic surveys suggest frequent sexual reproduction. Using microsatellite data from over 580 larvae from 13 colonies, we demonstrate that *P. damicornis* displays a mixed reproductive strategy where sexual and asexual larvae are produced simultaneously within the same colony. The majority of larvae were parthenogenetic (94%), but most colonies (10 of the 13) produced a subset of their larvae sexually. Logistic regression indicates that the proportion of sexual larvae varied significantly with colony size, cycle day, and calendar day. In particular, the decrease in sexual larvae with colony size suggests that the mixed reproductive strategy changes across the life of the coral. This unique shift in reproductive strategy leads to increasingly asexual replications of successful genotypes, which (in contrast to exclusive parthenogens) have already contributed to the recombinant gene pool.

## Introduction

Evolutionary theory predicts that sex is beneficial because recombination generates new, potentially advantageous gene combinations (Weismann 1886; Fisher 1930; Muller 1932) and helps to purge deleterious alleles (Muller 1964; Kondrashov 1988) by exposing them to natural selection (Hill and Robertson 1966; Felsenstein 1974). In contrast, asexual reproduction propagates successful genotypes and does not require mates (Weismann 1886; Williams 1975; Holman 1987; Radtkey et al. 1995; Weider et al. 1999). Most asexual lineages depend on occasional sexual recombination to add variation to their genomes, purge deleterious mutations, and adapt to changing environments. A mixed strategy of asexual propagation with low levels of sex combines the best of both strategies (Hurst and Peck 1996). For example, sperm-dependent parthenogenesis allows for recombination through occasional leakage of parental DNA from con- or heterospecific sexual progenitors (Beukeboom and Vrijenhoek 1998). Sexual and parthenogenetic reproduction are usually well separated by time (e.g., in cyclic parthenogens) or population (geographic parthenogens) or produce different offspring (e.g., in many insects).

Most reef-building corals reproduce sexually and asexually. Sexual reproduction is achieved by either free spawning eggs and sperm, or internally brooding larvae inside the coral polyp (Harrison 2011). The resulting planula larvae then disperse, settle, and metamorphose to form a new coral polyp. In colonial corals, the colony then grows by asexual fission of individual polyps. Some corals can reproduce asexually via vegetative fragmentation (Highsmith 1982). In a handful of coral species, genetically identical coral colonies can also be formed through the production of parthenogenetic larvae (Harrison 2011). Parthenogenesis was first documented in the Indo-Pacific coral *Pocillopora damicornis* (Stoddart 1983), where it has been confirmed repeatedly using allozyme data (Stoddart 1986; Ayre and Miller 2004; Sherman et al. 2006). None of these studies detected any sign of sexual reproduction, even though population genetic surveys suggest frequent sexual recombination amidst predominantly asexual reproduction (Ayre and Miller 2004). Recent microsatellite analyses indicate that two *P. damicornis* colonies in Taiwan produced a subset (6% and 29%) of their larvae sexually (Yeoh and Dai 2010), suggesting that *P. damicornis* can simultaneously produce asexual and sexual larvae. The exact origin of the larvae remains unclear. Several authors hypothesized that asexual larvae originate via budding, analogous to polyp budding (Muir 1984; Stoddart and Black 1985; Ward 1992); however, histological observations of gametogenesis and embryogenesis suggest that parthenogenetic larvae are the result of vegetative embryogenesis in diploid oocytes (Harriott 1983; Martin-Chavez 1986; Diah-Permata et al. 2000).

The evolutionary significance of parthenogenetic reproduction in *P. damicornis* remains unknown. The extent and patterns of parthenogenetic larvae production among colonies are poorly defined and nothing is known about the potentially different roles and fates of sexual versus parthenogenetic larvae. Using microsatellite genotyping, we quantified the extent of sexual and asexual reproduction across multiple colonies of *P. damicornis*. We examined whether colony genotype, size, or habitat is associated with the production of parthenogenetic and/or sexual larvae. In addition, we asked if parthenogenetic and/or sexual larvae are preferentially released at particular times during the lunar and/or reproductive cycle, to characterize potential differences (e.g., dispersion vs. population maintenance – sensu Stoddart 1988).

## Materials and Methods

### Organisms and larvae collection methods

**Figure 1:**
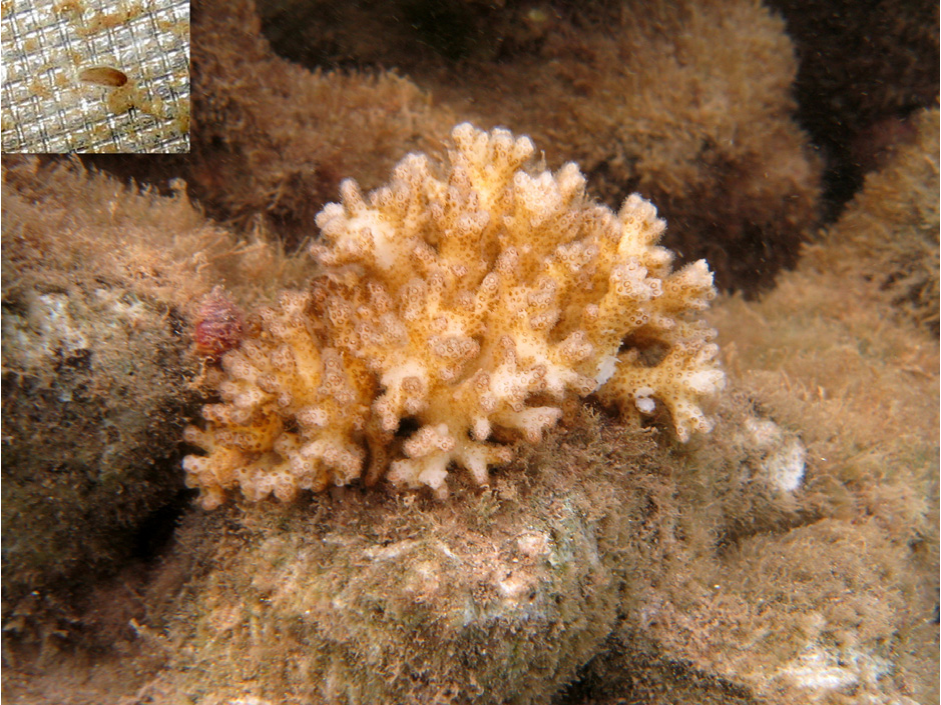
A *Pocillopora damicornis* colony on the reef in front of the Gump Research Station in Moorea, French Polynesia. The small insert shows a *P. damicornis* larvae on a mesh collection filter (100 μm).

In January 2010, 23 *P. damicornis* colonies (Fig. 1) were collected from fringe and back reef sites on the north shore of Moorea, French Polynesia. Colonies were transported to the Gump Research Station and kept in large seawater tanks (approximately 5 m^3^). A small nubbin (1 cm) was sampled from each colony and preserved in Guanidinium-Isothiocyanate (GITC) buffer for genetic analysis. Three days before the new moon (January 15th and February 13th), colonies were transferred to individual containers with continuous seawater inflow. The outflow of each container was diverted into a short PVC tube with a mesh filter (100 μm), where the larvae accumulated during planulation (Fig. 1). Larvae were collected using a 1000-μL micropipette and preserved individually in 200-μL GITC buffer (Fukami et al. 2004). Between January 17 and 20, colonies were monitored throughout the night from 19:00 to 06:00 h and larvae were collected and preserved as frequently as possible (i.e., every 2–3 h). In February 2010, planulation lasted from February 14 to 26, that is, until two nights before the full moon, and larvae were collected only in the morning.

### Parent colonies

*Pocillopora damicornis* were collected from fringing and back reef habitats to assess potential environmental influences on reproductive strategies. *Pocillopora damicornis* is very abundant on fringe reefs around Moorea, whereas on back reefs it is restricted to wave-protected grooves and crevices.

Colony size is often related to colony age and fecundity (Harrison 2011). Its impact on reproduction was measured in two ways. Colony size (as plane surface area, based on the longest and shortest colony diameter) was included in the main logistic regression analyses as a continuous variable. In addition, a separate comparison of larvae production between large and small colonies was conducted. Small colonies (*n* = 7) were defined as having a maximum diameter of less than 10 cm and a surface area under 75 cm^2^, whereas large colonies (*n* = 4) had minimum diameter over 10 cm and a surface area over 130 cm^2^ (Table 2).

Eighteen of the 23 *P. damicornis* colonies collected released each between 1 and over 1000 larvae during our observations following the new moon in January and February 2010. In order to ensure reliable characterization of each colony's reproductive patterns, only colonies that released more than 10 larvae (*n* = 13) were included in the analyses presented here.

### Genetic analyses

DNA was extracted with Agencourt DNAdvance® isolation kits (Beckman Coulter Inc., Brea, CA). Ten microsatellite loci were amplified with primers developed by Magalon et al. (2004) and Starger et al. (2007). PCR products included infrared labels on forward primers, and were manually sized and scored on a LICOR 4300 Genetic Analyzer (Li-Cor Biosciences, Lincoln, NE). Six loci amplified consistently using modified PCR protocols (Combosch and Vollmer 2011) and were used in all subsequent analyses (Table 1). Only larvae that were genotyped successfully at five or more loci were included in the analyses. Linkage disequilibrium among loci was assessed using a Markov chain Monte Carlo resampling method (Guo and Thompson 1992) in genepop (Raymond and Rousset 1995), followed by sequential Bonferroni correction to account for multiple comparisons. Probability of identity (PI) and the inbreeding coefficient *F*_IS_ were calculated using genalex 6.4.1 (Peakall and Smouse 2006).

**Table 1:** Loci-specific statistics for each of the six sampled microsatellite loci

Locus	*n*	*N*(A)	PI	Sex	*F*_IS_
Pd1	585	2	0.38	5	−0.54*
Pd2	591	3	0.21	14	−0.42*
Pd4	558	3	0.20	12	−0.29*
Pd5	592	3	0.34	9	−0.36
Pd6	593	3	0.47	16	**0.10**
Pv6	545	6	0.15	11	−0.16
Total	596	20	3.9×10^−4^	67	−0.28

*n*, *N*(A) and PI are based on the full dataset. *N,* Number of samples; *N*(A), Number of Alleles; PI, Probability of Identity; Sex, Number of times the locus varied in sexual larvae; *F*_IS_, Inbreeding coefficient among unique colony genotypes.

* Significant deviation from HWE (*P*<0.05).

Larvae with genotypes identical to their parent were considered parthenogenetically produced. Larvae with genotypes that differed from the maternal genotype were considered sexually produced. Sexually produced larval genotypes were examined for signs of selfing (i.e., absence of alleles not already present in the maternal genotype).

### Data analyses

Logistic regression analyses were used because the data are dichotomous (i.e., sexual vs. asexual) and binomially distributed (Wilson and Hardy 2002; Warton and Hui 2011). The main logistic regression analysis was conducted using a generalized linear mixed-effects model with the proportion of sexually produced larvae per colony per day as the response variable. Six different predictor variables were included as fixed effects in the model. Three factors were colony-related, time-independent factors: colony genotype, colony size, and colony habitat. Three factors were time-related, colony-independent factors: calendar day, lunar day, and release day. Lunar day and release day were used to assess daily patterns of larval release across reproductive cycles (i.e., January and February 2010). Lunar day refers to days after the new moon, whereas release day refers to the day after a specific colony started to release larvae (i.e., days into the larval release cycle of a colony). “Colony ID” was treated as a random effect to determine whether the results were consistent across colonies.

A second logistic regression analysis was used to compare the proportion of sexually produced larvae between small and large colonies. The same type of generalized linear mixed-effects model was used with the two size categories as predictor variables. All statistical analyses were conducted in the program r version 2.14.0 (R Core Team 2012) using the glmer function in the lme4 package (Bates et al. 2009).

## Results

Over 3000 larvae were released by 18 *P. damicornis* colonies. One colony (P10), released more than 1000 larvae over just three nights in January 2010. In total, 960 larvae were collected and preserved in GITC buffer for microsatellite genotyping; 597 larvae were successfully genotyped at 5–6 microsatellite loci (Table 1). Data analyses were conducted on 583 larvae that were released by 13 colonies, which each released 10 or more larvae (Table 2).

**Table 2:** List of maternal colonies, including colony size, the total number of genotyped larvae per colony (No. Larvae), the number and the percentage of sexually produced larvae per colony (% and No. Sex, respectively)

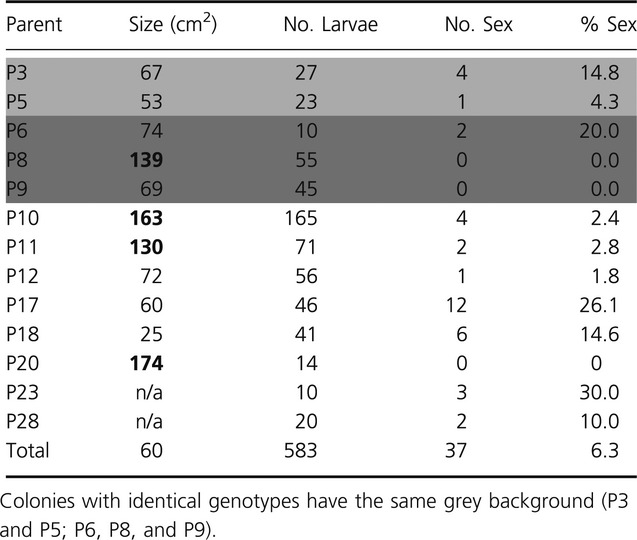

The six microsatellite loci had between 2 and 6 alleles (Table 1). No significant linkage disequilibria (LD) between loci were detected among colony genotypes (*P* > 0.05 after sequential Bonferroni correction). The PI to identify unique genotypes using all six loci was 3.9 × 10^−4^ (Table 1).

Of the 583 genotyped larvae, 546 larvae (93.7%) had identical genotypes to their parent and were scored as parthenogenetic larvae. The genotypes of the remaining 37 larvae (6.3%) differed from the parental genotype at one allele over one or more loci and were scored as sexual larvae. Eight of the 37 sexual larvae could have been produced via meiotic selfing; seven of these larvae had identical siblings, but differed from their brood parent's genotypes.

Ten of the 13 genotyped *P. damicornis* colonies released both sexually and asexually produced larvae (Table 2). All ten produced mostly parthenogenetic larvae (>70%). The other three colonies produced only parthenogenetic larvae (Table 2). Two of these three coral colonies had identical genotypes (P8 and P9). However, a third colony had the identical genotype as well (P6) and produced some larvae sexually. This suggests that parthenogenesis is influenced by environmental, demographic, and genetic factors.

### Reproduction changes with colony size and over time

Logistic regression analyses revealed significant effects of colony size, cycle day, and calendar day on the proportions of sexually produced larvae (Table 3). Colony genotype*,* colony habitat, and lunar day were not statistically significant (*P* > 0.05).

**Table 3:** Results of the generalized linear mixed effects model for the main logistic regression analysis

Predictor variable	χ^2^	df	*P*
Cycle day	8.36	1	0.004**
Colony size	4.74	1	0.029*
Calendar day	4.21	1	0.040*
Colony habitat	0.73	1	0.392
Colony genotype	0.44	1	0.505
Lunar day	0.03	1	0.873

Significance was determined using a Likelihood Ratio Test (LRT) χ.

** *P*<0.005, highly significant effects;

* *P*<0.05, significant effect.

**Figure 2:**
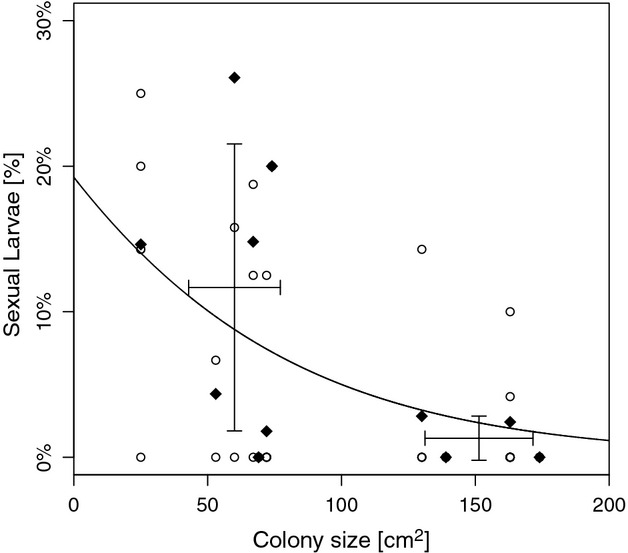
The proportion of sexually produced larvae decreases as a function of colony size (solid line). The average proportions of sexually produced larvae per colony are shown as black diamonds (♦), the average proportions per colony per cycle day are shown as white circles (o). Two different colony size classes are distinguished in this graph. Small colonies have a planar surface area under 75 cm^2^ and a maximum diameter of <10 cm (*n* = 7). Large colonies have a surface area over 130 cm^2^ (*n* = 4) and a minimum diameter of more than 10 cm. The cross on the left shows the average and the standard deviation for sexually produced larvae (and colony size) by small colonies. The cross on the right shows the significantly different results for big colonies (single-factor LRT: *P* = 0.02).

**Figure 3:**
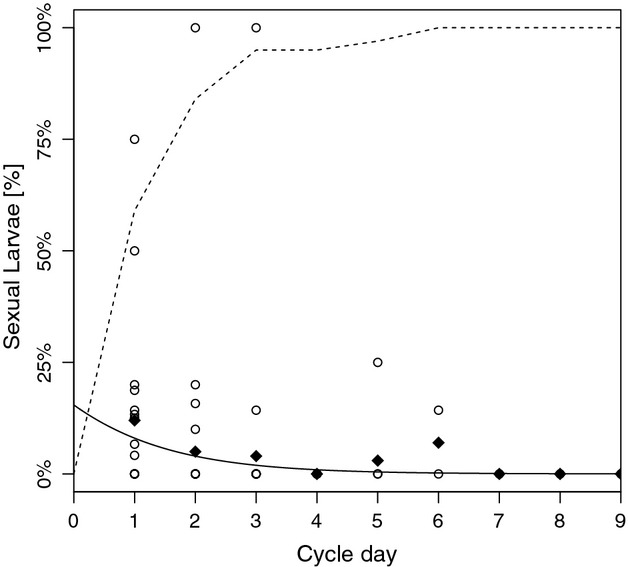
The proportion of sexually produced larvae released decreases during the reproductive cycles of *Pocillopora damicornis* colonies (solid line). The average proportions of sexually produced larvae per cycle day are shown as black diamonds (♦), the average proportions per cycle day per colony are shown as white circles (o). The cumulative percentage of sexual larvae across colonies is indicated by a dashed line (---).

The proportion of sexually produced larvae declined significantly with colony size (Likelihood Ratio Test [LRT], *P* = 0.029; Fig. 2). Moreover, small colonies produced significantly more larvae sexually (approximately 10.5%) than large colonies (approximately 2%; Table 2 and Fig. 2; single-factor LRT: *P* = 0.02). The proportion of sexually produced larvae also declined significantly over the course of each colony's larval release cycle (Cycle Day; Fig. 3; LRT, *P* = 0.004). However, the proportion of sexually produced larvae increased significantly over the 2-month monitoring period (calendar day; LRT, *P* = 0.040), for example, from January (7/223; 3.1%) to February (30/360; 8.3%).

## Discussion

Microsatellite data from 583 coral larvae and their 13 brood–parents indicate that *P. damicornis* in Moorea use a simultaneously mixed sexual and asexual reproductive strategy. Over two reproductive cycles, 10 of the 13 surveyed *P. damicornis* colonies produced both sexual and parthenogenetic larvae. All colonies produced on average 87% of their larvae parthenogenetically; three colonies produced only parthenogenetic larvae. The proportion of sexual reproduction decreased significantly with increasing colony size and during each colony's individual larval release cycle. However, over the bimonthly monitoring period, sexual reproduction increased. Parental genotype, habitat, and lunar day did not have significant effects on the proportion of sexual larvae. Although the exact mechanisms of this strategy remain unknown, it appears that *P. damicornis* applies a minimal sex strategy, dominated by asexual parthenogenesis.

Previous genetic analyses of *P. damicornis* did not detect any sexually produced larvae (Stoddart 1983, 1986; Ayre and Miller 2004; Sherman et al. 2006). Sexually produced larvae might have been missed due to their low frequency (approximately 6%), or misidentified as parthenogenetic due to the low resolution of allozyme markers. In contrast, allozyme-based population genetic surveys of *P. damicornis* provided evidence for sexual recombination (i.e., high genotypic diversity) in *P. damicornis* populations across the Central-West Pacific (Western Australia: Stoddart 1984a,b; Whitaker 2006; Great Barrier Reef: Benzie and Williams 1995; Ayre et al. 1997; Ayre and Miller 2004; Sherman et al. 2006; Lord Howe Island: Miller and Ayre 2004; Okinawa: Adjeroud and Tsuchiya 1999; and potentially in Hawaii: Stoddart 1986). Histological studies of *P. damicornis* in Taiwan and Hawaii also suggested regular embryogenesis and found no indications of asexual budding (Martin-Chavez 1986; Diah-Permata et al. 2000). Recently, Yeoh and Dai (2010) used multilocus microsatellite markers to compare larvae of two *P. damicornis* colonies in Taiwan and documented asexual and sexual larvae release. Both histologic and indirect and direct genetic studies thus indicate that *P. damicornis* reproduces mixed, sexually and asexually, throughout the Central-West Pacific.

### Mixed reproduction

One of the most intriguing characteristics of the mixed reproductive strategy of *P. damicornis* is the observed simultaneous release of parthenogenetic and sexual larvae. Simultaneous mixed reproduction is rare in animals. In most animals that display mixed reproduction, sexual and asexual reproduction, are separated by different conditions. In cyclical parthenogens such as monogonont rotifers, aphids, and *Daphnia*, sexual reproduction is limited to particular seasons, environmental conditions and/or population densities (Hebert 1974; Gómez and Carvalho 2001; Simon et al. 2002). In geographic parthenogens like squamate lizards and grasshoppers, asexual reproduction occurs in isolated populations, at particular high altitudes and latitudes or at the margins of species ranges (Vrijenhoek and Parker 2009). Animals that reproduce simultaneously sexually and asexually generally produce parthenogenetic offspring that are either haploid (e.g., hymenoptera) or predominantly homozygous (e.g., cockroaches and termites). *Pocillopora damicornis* are somewhat distinct in that sexual and apomictic parthenogenetic reproduction occurs simultaneously within the same coral colony.

Recent histological examinations clearly indicate that *P. damicornis* larvae result from oocytes following regular embryogenesis (Diah-Permata et al. 2000). As parthenogenetic larvae have identical genotypes, including several heterozygotic loci, they must stem from apomictic diploid eggs. But haploid eggs are required for sexually produced larvae. This suggests that both haploid and diploid eggs would have to be produced; yet no indications of distinct types of oocytes and/or eggs have ever been reported in *P. damicornis*.

Alternatively, eggs might be generally diploid and the maternal chromosome constitution is retained or restored postfertilization in parthenogenetic larvae. For example, in pseudogametic taxa, parthenogenetic reproduction requires sperm to stimulate embryogenesis without incorporating the parental DNA. Frequently, however, sperm nuclei evade complete degeneration and chromosome fragments, complete chromosome, or entire sperm genomes ‘leak’ into the parthenogenetic eggs (Schartl et al. 1995; D'Souza et al. 2006; D'Souza and Michiels 2009). The paternal chromosomes are then either added to the genome (chromosome addition) or the maternal ploidy level is restored by removing a maternal chromosome set. Thus, a predominantly apomictic parthenogenetic clutch would contain few genetically diverse propagules with small-to-moderate genotypic differences (D'Souza and Michiels 2009), which is exactly what we observed in *P. damicornis*.

Pseudogamy is rare, but occurs in a wide variety of taxa. It is more common among hybrid lineages, but nonhybrid pseudogametic taxa are also known (e.g., platyhelminthes, Beukeboom and Vrijenhoek 1998). As parental leakage occurs regularly in pseudogametic taxa, it has been termed “minimal sex” (Beukeboom and Vrijenhoek 1998) or “cryptic sex” (Schlupp 2005).

There are several striking similarities between the genetic patterns of mixed reproduction in *P. damicornis* and in the pseudogametic turbellid *Schmidtea polychroa* (Platyhelminthes). Two microsatellite studies of *S. polychroa* (D'Souza et al. 2004, 2006) report similar proportions of sexual offspring per parent (2–25% compared to 2–30% here) and across parents (6% each). Additionally, the number of loci affected by parental leakage (1–4 of 4) and the relative prevalence of parental leakage among fertile parents (6/11) is very similar to our results (1–5 of 6 loci and 10/13 fertile parents). Pseudogamy also explains the remarkable synchrony of asexual and sexual reproduction.

A common consequence of parental leakage in pseudogametic taxa is temporary polyploidy (Beukeboom and Vrijenhoek 1998). In *P. damicornis* larvae*,* polyploid allozyme genotypes have been described previously (Stoddart 1986) and several sexually produced larvae had multiple alleles at individual loci in this study (i.e., two maternal and one paternal). However, in other cases sexual reproduction led to a decrease in the number of observed alleles, from heterozygous in the colony to homozygous in the larvae. The reproductive patterns observed in *P. damicornis* thus match patterns described in pseudogametic taxa. Because most genotypic distances between maternal and larval genotypes were small (1 or 2 loci), it is possible that somatic mutations and/or chimeric colonies (reviewed in van Oppen et al. 2011) may have contributed to the signature of sexual reproduction.

### Evolutionary significance

Mixed reproduction is often described as a best-of-both-worlds scenario (Hurst and Peck 1996; Peck and Waxman 2000). It enables effective propagation of successful genotypes via parthenogenesis while sexual recombination constantly generates offspring with new allele combinations for selection. The extent of these benefits depends on the frequency of sex. In many cases a predominantly asexual strategy with a low frequency of sex seems to be most effective (Hurst and Peck 1996; Peck and Waxman 2000).

Mixed reproduction conveys many evolutionary advantages. In organisms using mixed reproductive strategies, natural selection acts not only on the level of the entire genome, as in self-replicating asexual taxa, but also on the level of individual genes, which are exposed to natural selection due to occasional sexual recombination. Moreover, the extent of asexual and sexual reproduction could be fine-tuned among genotypes so that successful genotypes are replicated mostly intact while less successful genotypes are more involved in sexual recombination.

Mixed reproduction can help organisms adapt to changing environments. The extent of asexual and sexual reproduction may vary across environments. If environmental conditions deteriorate and organisms become stressed, mixed reproducers might be capable of increasing the proportion of sexual reproduction. Increased sexual recombination would generate a higher proportion of novel genotypes and a more diverse cohort of offspring to deal with the new condition.

Another benefit of mixed reproduction is reproductive assurance. Reproductive failure because of sperm limitation is a serious problem for many broadcast sperm–dependent marine invertebrates (Yund 2000). Parthenogenesis assures reproduction independent of fertilization success. Moreover, if unfertilized eggs of pseudogametic taxa are capable of eventually developing into parthenogenetic larvae without pseudofertilization, a second tier of parthenogenetic larvae would start to develop and consequently be released slightly delayed. This is consistent with the observed decrease in the proportion of sexual larvae during the reproductive cycle of individual colonies.

### Sexual reproduction decreases with increasing colony size

Among the most remarkable characteristics of *P. damicornis's* mixed reproduction is that bigger colonies reproduce more asexually than smaller colonies (Fig. 2). Because the proportion of sexual reproduction was genotype and habitat independent, the decrease in sexual reproduction with colony size appears to be a general life-history strategy. Colony size is an important proxy for a colony's fecundity and evolutionary fitness (Hughes et al. 1992). Correlations between colony size and reproductive strategy therefore have important evolutionary consequences. Large colonies affirm the success of their genotypes in local environments. The shift toward increasingly asexual reproduction by larger colonies could thus lead to increased recruitment and survival of these successful genotypes in larval cohorts. However, in contrast to fully parthenogenetic taxa, successful brood parents will have already contributed “good alleles” to the gene pool when they were small. Over time, this reproductive strategy should lead to a highly inbred population structure with moderately high genotypic diversity (due to ongoing sexual recombination). The genotype distribution should consist of a few successful genotypes, present in multiple colonies (but particularly dominant among big colonies) and many subordinate genotypes. This is exactly what has been observed in population genetic and demographic studies, not only in French Polynesia (C. Marks, D. Combosch and S. Vollmer, unpubl. ms.) but also in other *P. damicornis* populations across the Central-West Pacific (Stoddart 1984b; Whitaker 2006).

### Sexual reproduction decreases during colonies' reproductive cycle

Sexually produced larvae were released significantly earlier during the reproductive cycle of individual colonies than asexually produced larvae. One possible reason is that unfertilized larvae eventually develop into parthenogenetic larvae, as outlined above (reproductive assurance hypothesis). Alternatively, sexually produced larvae might simply develop faster than their asexual counterparts, as is commonly observed in other taxa, for example, in termites (Matsuura 2010) and cockroaches (Corley and Moore 1999). Parental factors might stimulate the development of sexual offspring, as in *Drosophila melanogaster* (Yasuda et al. 1995) and *Caenorhabditis elegans* (Browning and Strome 1996). Faster development of sexual offspring could also be mediated genetically, for example, via heterosis. Ultimately, sexually produced larvae might simply be preferred by the mother, as a means to allocate resources in a way that maximizes overall offspring fitness (Michiels et al. 1999).

### Polyembryos

Three different colonies (P3, P6, and P17) released sexually produced, but genetically identical siblings (two pairs of twins and one triplet). One pair of genetically identical, sexually produced *P. damicornis* larvae had also been observed in Taiwan (Yeoh and Dai 2010). Genetically identical, sexually produced twins result from a single zygote that eventually divides into two or more clonal polyembryos, akin to monozygotic twins in humans. This form of limited polyembryony is considered accidental and does not seem to bear much evolutionary significance (Craig et al. 1997). While most *P. damicornis* polyps tend to produce only one larva at the time, multiple larvae per polyp are not uncommon (Stoddart and Black 1985; Martin-Chavez 1986). Moreover, viable settlement-competent polyembryos are known from other coral species, where free-swimming embryos break apart and multiply (Heyward and Negri 2012).

## Conclusion

The results of this study in combination with Yeoh and Dai (2010), and several population genetics studies throughout the Central-West Pacific, showed that asexual and sexual larvae brooding is the predominant reproductive strategy of *P. damicornis* populations in the Central-West Pacific. Populations at the margins of the species distribution range reproduce by broadcast spawning, for example, at the Southern GBR (Schmidt-Roach et al. 2012), in the Tropical Eastern Pacific (Glynn et al. 1991; Chávez-Romo and Reyes-Bonilla 2007) and in Western Australia (Stoddart and Black 1985; Ward 1992), although taxonomic uncertainties exist for some of these peripheral populations (Schmidt-Roach et al. 2013). Multiple *P. damicornis* types with reproductive differences have been confirmed in Hawaii (Richmond and Jokiel 1984) and inferred in Western Australia (Ward 1995) and the Western Indian Ocean (Souter 2010). In addition, interspecific and intergeneric hybridization was inferred for peripheral *P. damicornis* populations in the Tropical Eastern Pacific (Combosch et al. 2008) and at Lord Howe Island (Miller and Ayre 2004). In light of the confusing, sometimes contradictory results of allozyme-based studies, it is now clear that more high-resolution data (microsatellites, SNPs) are needed to assess the spatial and temporal variability in sexual reproduction and the impact of environmental variation on the reproductive strategy of *P. damicornis*. Eighty years after the first description of the reproductive strategy of *P. damicornis* (Marshall and Stephenson 1933) a lot of basic questions remain to be answered.
